# Fine Control of Optical Properties of Nb_2_O_5_ Film by Thermal Treatment

**DOI:** 10.3390/mi15121453

**Published:** 2024-11-29

**Authors:** Xianpeng Liang, Bowen Liu, Quan Yuan, Xiaomin Lin, Shaopeng Ren, Shuaifeng Zhao, Xiaojun Yin, Shuguo Fei

**Affiliations:** Shenyang Academy of Instrumentation Science, Shenyang 110043, China

**Keywords:** thermal treatment, optical thin film, residual stress, thermal stability, optical properties

## Abstract

Thermal treatment is a common method to improve the properties of optical thin films, but improper thermal treatment processing will result in the degradation of the optical properties of the optical thin film. The thermal stability of niobium oxide (Nb_2_O_5_) thin films prepared by magnetron sputtering was systematically studied by analyzing the roughness and morphology of the film under different thermal treatment processes. The results show that the amorphous stability of the Nb_2_O_5_ thin film can be maintained up to 400 °C. Before crystallization, with an increase in annealing temperature, the surface roughness of the film has no obvious change, the refractive index decreases, and the elastic modulus and hardness increase. The residual stress was measured by a laser interferometer. The results show that the residual compressive stress is present in the film, and the residual stress decreases with an increase in thermal treatment temperature. Considering the residual stress state, phase composition, mechanical properties, and optical properties of Nb_2_O_5_ films at different thermal treatment temperatures, we believe that the spectral position of the optical thin film device can be finely controlled within a 1.6% wavelength, and the thermal treatment temperature of Nb_2_O_5_ films prepared by magnetron sputtering should not exceed 400 °C.

## 1. Introduction

With the continuous development of modern optical technology, optical coating technology has gradually penetrated into various fields, such as imaging, space exploration, and industrial production [1]. By alternately depositing films with different thicknesses and characteristics, optimization and control of the performance of optical devices, including anti-reflection, high reflection, spectral division, and other functions, can be achieved. The rapid advancement of optical technology propels optical coatings to continually progress into more extreme areas, including ultra-thin layers, ultra-thick layers, and super-multilayers. Undoubtedly, this poses new challenges for the design and preparation of optical coatings [2,3,4,5].

Physical Vapor Deposition (PVD) techniques are at the forefront of optical coating deposition, offering precise control over coatings’ thickness and composition. These techniques include thermal evaporation, electron beam evaporation, magnetron sputtering, and ion beam sputtering [6,7,8,9]. Magnetron sputtering stands out as a versatile and widely employed PVD technique with unique characteristics, which is widely used in various application fields such as metal, alloy, composite material, functional film, and optical coatings because of its high deposition rate, good uniformity, high film density, lower film pollution, strong film adhesion, good repeatability, and other excellent technical characteristics [10,11,12,13,14].

In the realm of intricate precision applications, employing appropriate post-processing techniques becomes imperative for enhancing their performance. Thermal treatment serves as a widely adopted approach for enhancing the performance of optical coatings by facilitating atomic diffusion while alleviating residual stress and defects within the films [9,15,16,17,18,19]. Nevertheless, optimizing the process of thermal treatment becomes crucial due to potential drawbacks such as heightened surface roughness and aggravated surface properties resulting from elevated temperatures. Moreover, distinct thermal treatment methods must be devised for diverse substrates and films so as to avoid detrimental impacts on optical properties arising from amorphous material crystallization at high temperatures.

Nb_2_O_5_ films find widespread application in contemporary optical and microelectronics technology owing to their exceptional performance, spanning across optical coatings, electrochromic devices, gas sensors, catalysis, photoelectrodes, and high-energy-density capacitors [20]. Among the five stable compounds of niobium and oxygen (NbO, NbO_2_, Nb_2_O, Nb_6_O, and Nb_2_O_5_), Nb_2_O_5_ stands out as the most stable compound with a high refractive index and low absorption coefficient. Consequently, it serves as an ideal material for optical coatings within the visible-near-infrared band. Moreover, Nb_2_O_5_ exhibits various crystal structures including pseudo-hexagonal (TT-Nb_2_O_5_), orthogonal (T-Nb_2_O_5_), and monoclinic (H-Nb_2_O_5_). These crystal structures can undergo transformations at different temperatures leading to variations in their physical and chemical properties. Numerous studies have been conducted on the properties of Nb_2_O_5_ thin films [10,12,17,21,22,23]; however, the focus has primarily been on investigating the influence of process parameters during film deposition rather than conducting comprehensive theoretical research on the effects of thermal treatment processes on coatings’ properties.

In this work, we focused on the growth process and thermal stability of Nb_2_O_5_ amorphous films prepared by magnetron sputtering. We hope to guide the design process of optical coatings by studying the characteristics of different stages of film growth, so as to avoid the appearance of low-quality films in the design. At the same time, the thermal stability of the Nb_2_O_5_ film and the change in film properties with temperature are studied to guide the heat treatment processing of the film. By examining Nb_2_O_5_ films with varying thicknesses, we were able to determine their morphological characteristics at different stages of growth. Furthermore, an analysis was conducted to understand the impact of thermal treatment on various aspects such as optical properties, mechanical properties, residual stress, chemical composition, and phase composition of Nb_2_O_5_ amorphous films and the underlying reasons for these changes. The results demonstrate a noticeable enhancement in the overall performance of Nb_2_O_5_ amorphous films as the thermal treatment temperature increases; however, it is advisable not to exceed 400 °C.

## 2. Materials and Methods

Glass and NaCl single crystals (<100>) were selected as substrates for Nb_2_O_5_ film deposition. The Nb_2_O_5_ films grown on a glass substrate were used to analyze the optical and mechanical properties of the film, and the films deposited on a NaCl single-crystal substrate were only used for DSC analysis. The glass substrate was washed thoroughly with water and ethanol in turn and then put into the coating machine after drying. The NaCl substrate was not processed.

Nb_2_O_5_ films were prepared using plasma-assisted reactive magnetron sputtering (Helios 400, Leybold, Alzenau, Germany). The parameters during deposition are shown in Table 1, which achieved a deposition rate of 0.5 nm/s. Subsequently, the deposited Nb_2_O_5_ films underwent annealing in air at temperatures ranging from 100 °C to 500 °C. Comprehensive investigations were conducted on changes in residual stress, spectral properties, optical constants, surface roughness, phase composition, and film thickness before and after annealing. Nb_2_O_5_ films were correspondingly denoted as Nb-t, where “t” is deposition time. For example, Nb-100 is the Nb_2_O_5_ film that underwent deposition for 100 s.

An ultraviolet–visible spectrophotometer was employed to measure the spectral properties of Nb_2_O_5_ films, while an ellipsometer was utilized for analyzing the optical constants. The phase composition of Nb_2_O_5_ films before and after thermal treatment was determined using grazing-incidence X-ray diffraction (XRD, Rigaku D/max-2400, Rigaku, Tokyo, Japan) with copper Kα (λ = 0.154178 nm) as the radiation source within a test range of 10°~80°. Scanning electron microscopy (SEM, LEO, SUPRA35, Ammerbuch, Germany) was employed to observe the surface and cross-section morphology, and statistical analysis of cross-section photos provided information on Nb_2_O_5_ film thickness. Atomic force microscopy (AFM, Dimension Icon, Bruker, Germany) measured roughness information in a test area measuring 5 μm × 5 μm. X-ray photoelectron spectroscopy (XPS, Thermo Fisher Scientific, Waltham, MA, USA), with Al as the radiation source and calibration using a C *1s* peak, determined the chemical state of elements on the surface of Nb_2_O_5_ films. Transmission electron microscopy (TEM, FEI, Tecnai G2 F20, Hillsboro, OR, USA), with an acceleration voltage of 200 kV, observed both the interface bonding state between the Nb_2_O_5_ film and substrate as well as crystallization changes in the film following annealing. The spectral properties were tested with a spectrophotometer (UV-vis, Cary7000, Agilent, Santa Clara, CA, USA), and the optical constants were measured with an ellipsometer (J. A. Woollam V-VASE, Lincoln, NE, USA).

## 3. Results and Discussion

### 3.1. Growth of Nb_2_O_5_ Film

**Figure 1:**
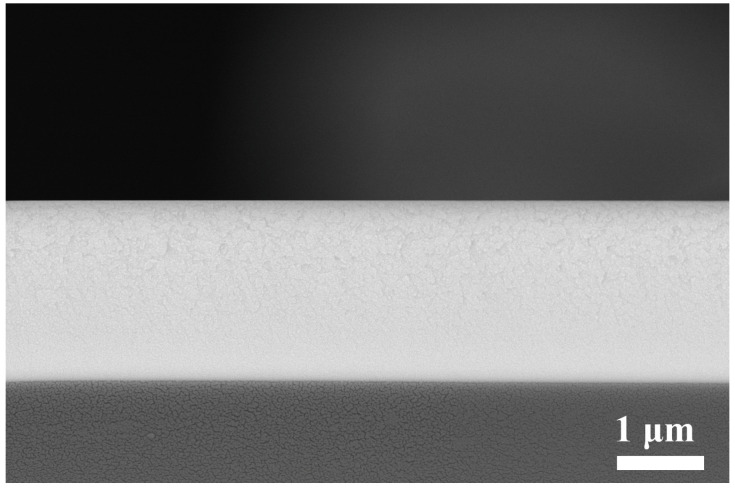
Microstructure of Nb_2_O_5_ film roughness.

To investigate the microstructure of Nb_2_O_5_ film at different growth stages more clearly, the cross-sectional morphology of Nb-4000 was examined using SEM, as shown in Figure 1. The observations reveal that at various growth stages, the film exhibits a uniform and dense structure without any visible pores. Moreover, the Nb_2_O_5_ film prepared in this study demonstrates an isotropic structure without distinct columnar features, indicating a well-bonded interface between the film and substrate. Typically, films grown by PVD tend to exhibit columnar structures due to the shadow effect and impaired diffusion capacity [23,24]. In this work, the movement of the substrate relative to the target significantly reduces the shadowing effect. Additionally, the close proximity between the target and substrate enables the film atoms to reach the substrate with higher energy. The bombardment of the substrate by the plasma also imparts additional energy to the film, which can reduce surface roughness and enhance uniformity.

**Figure 2:**
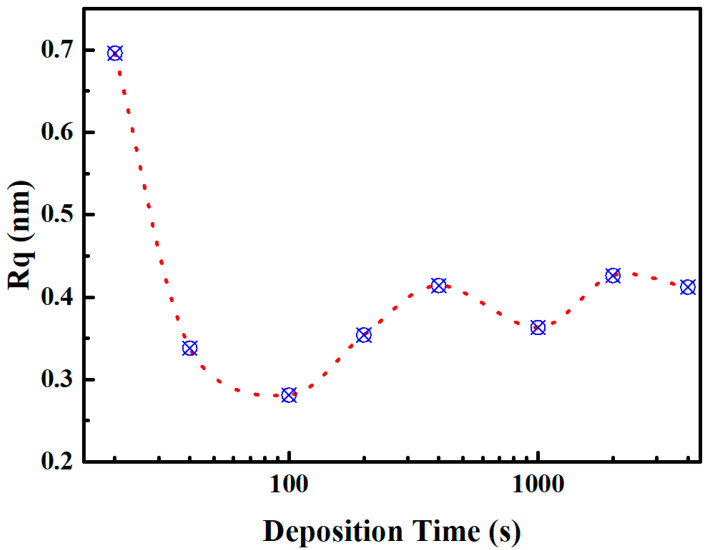
Nb_2_O_5_ film roughness changes with deposition time.

To further investigate the growth characteristics of the Nb_2_O_5_ film at different stages, films deposited for varying durations were characterized using AFM. The evolution of film roughness with deposition time was analyzed, as depicted in Figure 2. The substrate exhibits a roughness of 0.34 nm. In the early stages of film growth, the surface roughness undergoes significant changes, reaching a maximum at 20 s, with an Rq of approximately 0.7 nm. As the growth time extends to 100 s, the film exhibits the lowest roughness, with an Rq of around 0.28 nm. Subsequently, as the deposition time increases, the film roughness gradually stabilizes at approximately 0.35 nm, comparable to the initial roughness of the substrate. During the initial stages of film growth, the film atoms nucleate non-uniformly on the substrate surface in an island-like pattern, resulting in a surface roughness greater than that of the substrate. With prolonged deposition times, the film progressively achieves complete coverage of the substrate, causing the surface roughness of the film to replicate that of the substrate. Therefore, as the deposition experiment continues, the roughness of the film gradually stabilizes around the initial roughness of the substrate.

### 3.2. Structure Changes

Optical coatings are commonly engineered with amorphous structures to minimize scattering losses caused by misalignment between grain boundaries and grains. From a thermodynamic perspective, amorphous structures exhibit intrinsic instability, possess high free energy, and are susceptible to crystallization when exposed to high temperatures. Crystallization not only has the capability to alter optical characteristics but also impacts properties such as surface roughness and bonding strength in films. Consequently, it becomes crucial to thoroughly investigate the thermal stability of precision optical coatings due to their requirement for stress relief annealing during manufacturing processes as well as potential exposure under extreme conditions like intense laser irradiation during operation.

**Figure 3:**
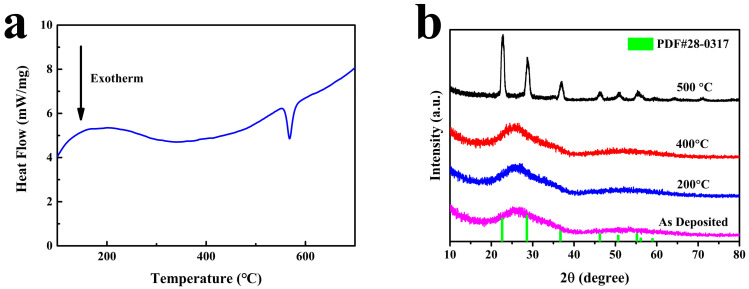
(**a**) DSC of Nb_2_O_5_ film; (**b**) Phase composition of Nb_2_O_5_ film at different temperatures.

Nb_2_O_5_ film powder was obtained by dissolving the NaCl substrate with water. The collected powder was dried and analyzed by DSC, and the heating rate was 10 °C/min, as shown in Figure 3a. We can see that the Nb_2_O_5_ film starts crystallizing at about 550 °C. Therefore, the experimental temperature of the thermal treatment we chose was not higher than 500 °C. Phase composition analysis of Nb-4000 annealing at different temperatures is presented in Figure 3. The initially prepared film exhibited a typical amorphous structure. When the annealing temperature was below 400 °C, the film maintained its amorphous structure, with no appearance of diffraction peaks in the XRD pattern. However, at an annealing temperature of 500 °C, sharp diffraction peaks began to emerge in the XRD pattern, indicating the initiation of crystallization within the film. Upon comparison with PDF cards, it was observed that the film gradually transformed from amorphous to a TT phase (JCPDS file No.: 28-0317; a = b = 3.607 Å, c = 3.925 Å, volume of the cell = 44.22 Å^3^). Regarding the impact of thermal treatment on the phase composition of Nb_2_O_5_ films, numerous studies suggest that the T and TT phases exhibit similar structures [20,21]. Distinguishing between these two phases is challenging. It has been reported that all refractive indices of the TT phase correspond to one or a pair of closely spaced peaks of the T phase [22]. Therefore, the TT phase is considered a poorly crystallized form of the T phase due to the highly similar crystal structures between these two phases [11,16]. There has been extensive research on the crystallization behavior of Nb_2_O_5_ films. Although there are some differences among the results of various studies, most research indicates that the crystallization temperature of Nb_2_O_5_ films is above 500 °C. The primary reason for the discrepancies lies in the different preparation techniques used for Nb_2_O_5_ films [20,21,22].

**Figure 4:**
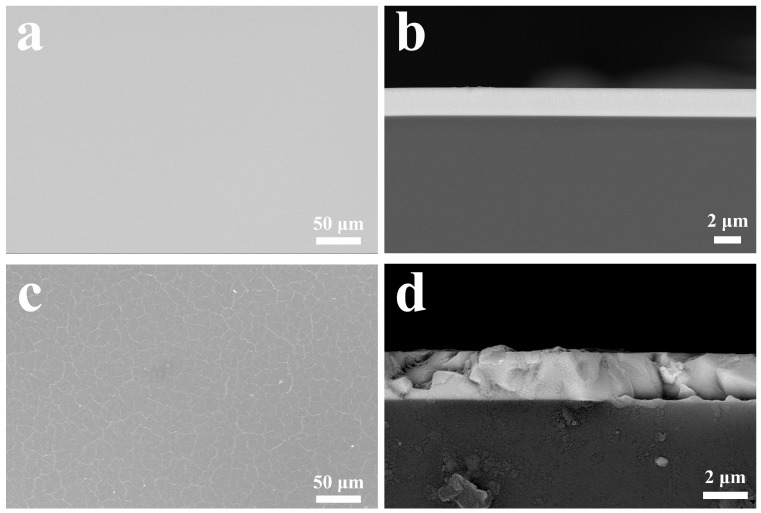
(**a**) Surface of Nb_2_O_5_ film annealed at 400 °C. (**b**) Cross-section of Nb_2_O_5_ film annealed at 400 °C. (**c**) Surface of Nb_2_O_5_ film annealed at 500 °C. (**d**) Cross-section of Nb_2_O_5_ film annealed at 500 °C.

The microstructure of the annealed films was examined, as illustrated in Figure 4. It is evident that when the annealing temperature is below 400 °C, there is no significant alteration in the surface and cross-sectional morphology, which remains consistent with the as-deposited state. However, as the annealing temperature increases and exceeds 500 °C, the surface of the Nb_2_O_5_ film exhibits noticeable roughness and unevenness, resembling that of stone.

**Figure 5:**
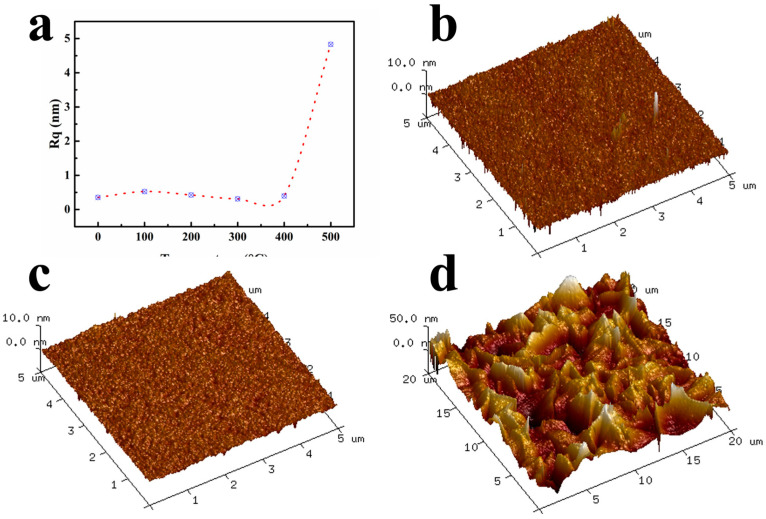
(**a**) Roughness changes in Nb_2_O_5_ film after annealing at different temperatures; (**b**) as deposited; (**c**) annealed at 400 °C; (**d**) annealed at 500 °C.

Roughness analysis of the Nb-200 film was performed using AFM after annealing at different temperatures, as shown in Figure 5a. Below 400 °C, the roughness remains relatively stable, approximately 0.4 to 0.5 nm, as shown in Figure 5b,c. When the temperature reaches 500 °C, the surface roughness increases dramatically to about 5 nm, as shown in Figure 5d. Below 400 °C, the film primarily undergoes uniform element diffusion and sintering processes without crystallization, resulting in minimal changes in roughness. However, as the annealing temperature rises to 500 °C, crystallization occurs within the film, leading to significant alterations and the formation of wrinkles on the film surface, which causes a rapid increase in surface roughness. Lai et al. [23] reached a similar conclusion, showing that once crystallization occurs in Nb_2_O_5_ films, the roughness increases sharply.

**Figure 6:**
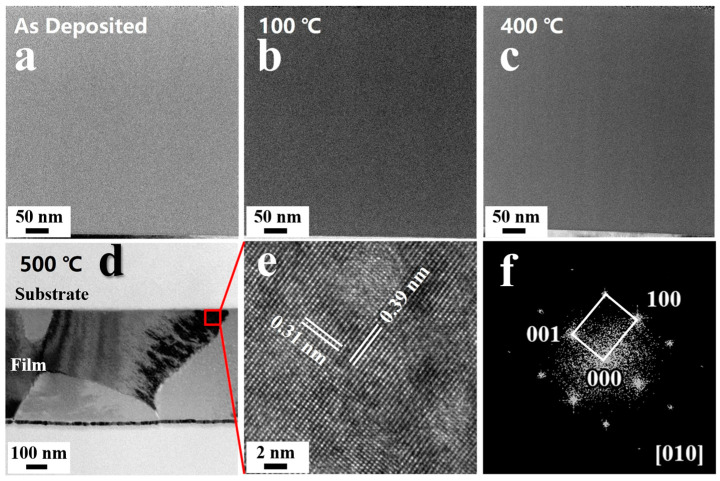
(**a**) Bright-field image of Nb_2_O_5_ film before annealing; (**b**) Bright-field image of Nb_2_O_5_ film annealed at 100 °C; (**c**) Bright-field image of Nb_2_O_5_ film annealed at 400 °C; (**d**) Bright-field image of Nb_2_O_5_ film annealed at 500 °C; (**e**) HRTEM of Nb_2_O_5_ film annealed at 500 °C; (**f**) SAED of Nb_2_O_5_ film annealed at 500 °C.

Observation of the microstructure of Nb_2_O_5_ films before and after annealing was conducted using TEM, as shown in Figure 6. It can be observed that when the temperature is below 400 °C, there is no significant change in the microstructure of the film. No columnar structures, pores, or crystallization are evident, indicating a relatively uniform film structure, which is consistent with the SEM analysis. Figure 6d shows the bright field of the Nb_2_O_5_ film annealing at 500 °C. We can see that as the temperature increases to 500 °C, crystallization occurs in the film. SAED results indicate that the film exhibits the TT phase, as it is shown in Figure 6e, consistent with the analysis from XRD. HRTEM analysis reveals a lattice spacing of 0.39 nm and 0.31 nm, corresponding to the (001) and (100) crystal planes of the TT phase.

### 3.3. Effect on Mechanical Properties

**Figure 7:**
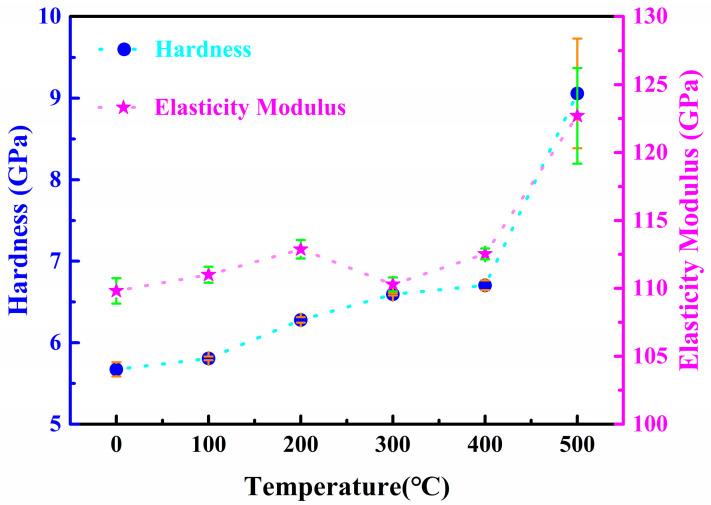
Hardness and elasticity modulus of Nb_2_O_5_ film annealed at different temperatures.

The mechanical properties of optical coating devices are closely related to their performance and longevity; therefore, we conducted an analysis of the mechanical properties of Nb_2_O_5_ films before and after thermal treatment. The nanoindentation method was employed to measure the hardness and elastic modulus of the Nb_2_O_5_ films, with the results presented in Figure 7. The hardness exhibited a gradual increase with rising thermal treatment temperatures. Specifically, after annealing at 400 °C, the hardness of the Nb_2_O_5_ films rose from an initial value of 5.7 GPa to 6.7 GPa, representing a 17.5% increase. However, the elastic modulus of the Nb_2_O_5_ films did not show a significant increase within this temperature range, remaining in the range of 110–113 GPa. This can be attributed to the minor changes in the microstructure of the film during the annealing process. While the hardness of the material is closely linked to its microstructure, the elastic modulus is more associated with the bonding modes between the atoms within the material, resulting in a less pronounced connection with the microstructure. Upon increasing the annealing temperature to 500 °C, both the hardness and elastic modulus of the film significantly improved. This enhancement is attributed to crystallization, which not only alters the microstructure of the film layer but also modifies the atomic bonding modes within the niobium oxide film. Goldenberg et al. [24] obtained similar results, showing that after crystallization, the hardness and Young’s modulus of the film significantly increase.

**Figure 8:**
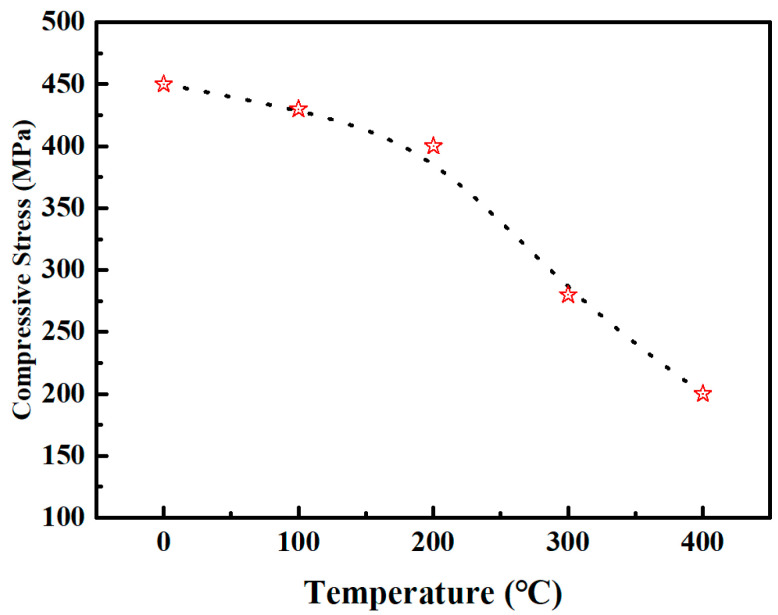
Residual stress of Nb_2_O_5_ film after annealing at different temperatures.

When the PVD method is employed for film deposition, the presence of residual stress is inevitable. Residual stress significantly impacts the surface figure accuracy of optical devices, influencing their performance in high-end precision applications. The Stoney formula calculates the residual stress in thin films by measuring the curvature change of the substrate. The simplified formula is as follows:

(1) $$\sigma =\frac{4E_{S}}{3(1-\nu_{S})}\frac{{t_{s}}^{2}}{t_{f}{D_{s}}^{2}}\Delta p$$

where *E_S_* and *ν_S_* represent Young’s modulus and Poisson’s ratio of the substrate, respectively; *t_s_* and *D_s_* denote the substrate’s thickness and diameter, respectively; *t_f_* is the film thickness; and *Δp* represents the change in the PV value before and after film deposition. Nb-4000 was selected as the research subject, and the PV values before and after annealing at different temperatures were measured to calculate the changes in residual stress. Figure 8 illustrates the variation in residual stress in the films at different annealing temperatures; we only studied the residual stress of Nb_2_O_5_ films before crystallization. As the annealing temperature increases, the residual stress in the film gradually decreases from approximately 450 MPa to about 200 MPa, a reduction of 55.6%, though the film remains in a state of compressive residual stress.

### 3.4. Changes in Optical Properties

**Figure 9:**
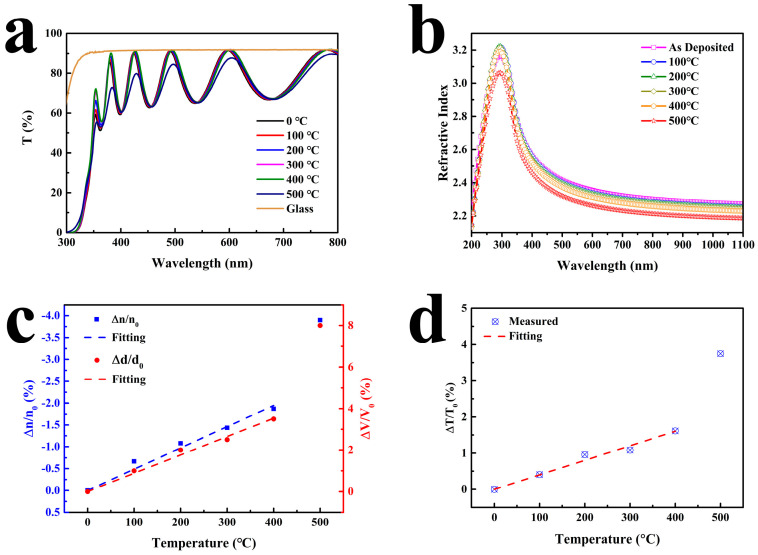
Optical properties of Nb-1000 annealed at different temperatures: (**a**) transmission spectra; (**b**) refractive index dispersion; (**c**) refractive index change rate at 550 nm and film volume change rate; (**d**) optical thickness change rate at 550 nm.

Changes in the optical properties of films have significant implications for the manufacturing processes and application environments of optical coating devices. Spectral performance tests were conducted on Nb-1000 after annealing at different temperatures, where the substrate is glass, as shown in Figure 9a. We can also see that the maximum transmittance of the coated glass is equal to that of the glass without coating, which indicates that the refractive index of the Nb_2_O_5_ film is uniform. It can be observed that when the annealing temperature is below 400 °C, the spectral positions of the sample gradually shift towards longer wavelengths, but the overall change is minimal. However, when annealed at 500 °C, not only does the spectrum exhibit a shift in position, but there is also a noticeable change in the transmittance at various peak positions.

In order to further analyze the reasons for the changes in the optical properties of the film, we conducted an in-depth characterization analysis using an ellipsometric spectrometer with a Cauchy model. The results are shown in Figure 9b. It can be observed that in the visible to near-infrared wavelength range, with an increase in annealing temperature, the refractive index (n) of the film gradually decreases, with the sample annealed at 500 °C exhibiting a particularly pronounced effect.

It is generally believed that the refractive index changes caused by annealing are related to the change in the film’s density [11]. An increase in the apparent volume of the film leads to a decrease in density, resulting in a reduction in the refractive index. Taking the refractive index at 550 nm as an example, the relationship between the refractive index, volume changes, and annealing temperature of the film is analyzed, as shown in Figure 9c. According to the fitting results, when the annealing temperature is below 400 °C, the refractive index of the film decreases linearly. When the annealing temperature reaches 400 °C, the refractive index of the film layer decreases by about 2%. The specific relationship is as follows:

(2) $$\frac{∆n}{n_{0}}=-0.00487T$$

The thickness of Nb_2_O_5_ films after annealing at different temperatures was also measured by ellipsometry. We can see that with an increase in annealing temperature, the thickness of the film gradually increases. When the annealing temperature rises to 400 °C, the film thickness increases by about 4%. After annealing at 500 °C, the thickness increases by 8%. Before 400 °C, the physical thickness of Nb_2_O_5_ film has a linear relationship with temperature:

(3) $$\frac{∆V}{V_{0}}=0.00883T$$

**Figure 10:**
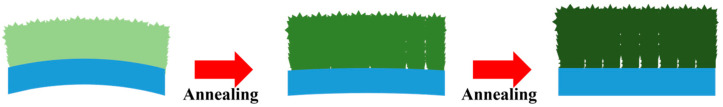
Effect of annealing on residual stress and thickness of film.

Significant efforts have been devoted to investigating the effects of thermal treatment on the performance of optical thin films, yet the research outcomes vary considerably [18,21,25,26]. A comparative analysis reveals that the impact of thermal treatment on film properties is not solely dependent on the thermal treatment process itself but is also closely related to the deposition techniques used and the intrinsic characteristics of the thin films. Different deposition processes result in varying film densities. If annealing increases the density of the films, the refractive index will increase accordingly; conversely, if annealing decreases the density, the refractive index will decrease [16,27,28,29]. In this work, the optical thickness of the film was calculated, and the results are shown in Figure 10. It can be observed that with increasing annealing temperature, the optical thickness of the film gradually increases, corresponding to the redshift of the spectrum mentioned earlier. Therefore, when designing optical thin films that undergo thermal treatment, a correction factor should be applied to Nb_2_O_5_ films.

It can be seen that before 400 °C, the optical thickness of the film also increases linearly with an increase in annealing temperature:

(4) $$\frac{∆T}{T_{0}}=0.00401T$$

When the annealing temperature rises to 500 °C, the refractive index of the film exhibits an abnormal decrease, accompanied by rapid volumetric expansion. This behavior corresponds to the crystallization of the film discussed earlier. Below the crystallization temperature, the volume change is mainly related to elemental diffusion and sintering within the film. The residual compressive stress inside the film causes a tendency for extrusion along the thickness direction. Therefore, as annealing progresses and the stress is released, the film thickness slightly increases and the density gradually decreases. Upon reaching the crystallization temperature, the crystallization process leads to more significant volume changes compared to diffusion and sintering, resulting in abrupt changes in both the volume and refractive index, as shown in Figure 10.

In addition, if the spectral positioning of the prepared optical thin film device deviates from the target specification, and the wavelength position is slightly short, with an error of less than 1.6%, adjustments can be made by thermal treatment without considering the changes in the properties of the low-refractive index material.

## 4. Conclusions

This study systematically investigated the performance and structural evolution of amorphous Nb_2_O_5_ thin films prepared by magnetron sputtering during the annealing process. In an air atmosphere, the amorphous structure of the films was maintained up to 400 °C; after annealing at 500 °C, the films transformed into the TT phase. Before crystallization, as the annealing temperature increased, the film thickness increased while the density decreased, leading to a reduction in the refractive index n. Before crystallization, the hardness of the films increased with increasing annealing temperature, while the elastic modulus remained largely unchanged. Prior to annealing, the films exhibited a state of compressive stress, which was progressively released as the annealing temperature increased, though a slight compressive stress was still retained overall. Based on these findings, it is recommended that the annealing temperature for amorphous Nb_2_O_5_ thin films should not exceed 400 °C.

## Figures and Tables

**Table 1:** Process parameters of Nb_2_O_5_ film deposition.

Parameters	PVD
Target	Nb
Argon Flow	35 SCCM
Oxygen Flow	40-55 SCCM
MF Power	4700 W
Temperature	180 °C

## Data Availability

Data will be made available on request.
